# Several Selections

**Published:** 1856-03

**Authors:** 


### SEVERAL SELECTIONS.

### QUACKOPATHY.

Take of Brandreth's pills,
A twenty-five cent box;
And of Townsend's Sarsaparilla,
Enough to kill an ox.

Before you go to bed,
Eat a quart of Salmagundi,
And on top of this,
Take a dose of "*alicomfundi.*"

Every night and morning,
Drink a pint of Brandy,—
Sweeten if you please,
With a stick of Cough Cure Candy.

Then add to the above,
A pail of Quacknip tea,—
Then if you are not dead,
You surely ought to be.

### SENSEOPATHY.

Take the open air—
The more you take the bette:
Follow nature's laws
To the very letter.

Let the doctors go
To the Bay of Biscay—
Let alone the Gin,
The Brandy and the Whisky,

Freely exercise:
Keep your spirits cheerful,
Let no dread of sickness
Make you over fearful.

Eat the simplest food,
Drink the pure cold water,
Then you will be well,
Or at least you ought ter.

